# DAY-TO-DAY CHANGES IN A SIMPLIFIED MEDITERRANEAN DIET: IMPLICATIONS FOR NEXT DAY COGNITIVE FUNCTION

**DOI:** 10.1093/geroni/igae098.4152

**Published:** 2024-12-31

**Authors:** Kelly Parker, Jeremy Hamm

**Affiliations:** North Dakota State University, Fargo, North Dakota, United States; North Dakota State University, Fargo, North Dakota, United States

## Abstract

Although individual differences in Mediterranean diet (MD) have been linked to better cognitive functioning over extended periods of time, little is known about day-to-day fluctuations in MD adherence and how it may influence daily cognitive functioning. This daily diary study examined a simplified Mediterranean diet (sMD) and its impact on cognitive functioning the following day over the course of one week. Participants (N=217, Mage = 54±15, 65% female) completed daily surveys and cognitive tasks over one week to measure their daily adherence to the sMD and their daily cognitive function. Lagged multilevel models assessed the impact of (within-person centered) daily sMD on next day cognitive functions that included episodic memory, executive functioning, and processing speed. Age, sex, income, education, and race were controlled in all models. Results showed that on days following higher than usual adherence to the sMD, participants had faster processing speed (b = -45.10, p < 0.01) and greater letter fluency (b = 0.49, p = 0.04) than on days after lower than usual sMD adherence. These findings show that day-to-day shifts in sMD adherence have implications for lagged changes in cognition for middle-aged and older adults, suggesting even small changes in sMD add up and could contribute to improved cognitive aging over time. Small, but simple changes such as increasing fruit, vegetable, and fish intake and reducing sugar-sweetened beverage consumption may thus result in improved cognitive functioning even over very brief time periods.

